# Editorial: Women in aquatic microbiology: 2022

**DOI:** 10.3389/fmicb.2023.1225575

**Published:** 2023-06-19

**Authors:** Annika Vaksmaa, Alessandra Adessi, Maria M. Sala, Alison Buchan, Catarina M. Magalhães, Adriane Clark Jones

**Affiliations:** ^1^Department of Marine Microbiology and Biogeochemistry, NIOZ Royal Netherlands Institute for Sea Research, Texel, Netherlands; ^2^Department of Agriculture, Food, Environment and Forestry, University of Florence, Florence, Italy; ^3^Departament de Biologia Marina i Oceanografia, Institut de Ciències del Mar, ICM (CSIC), Barcelona, Spain; ^4^Department of Microbiology, University of Tennessee, Knoxville, Knoxville, TN, United States; ^5^CIIMAR, Interdisciplinary Centre of Marine and Environmental Research, University of Porto, Matosinhos, Portugal; ^6^Department of Biology, Faculty of Sciences, University of Porto, Porto, Portugal; ^7^Department of Biological Sciences, Mount Saint Mary's University, Los Angeles, CA, United States

**Keywords:** women, STEM—Science Technology Engineering Mathematics, underrepresentation, aquatic ecosystems, microbiology

There is a notable underrepresentation of female scientists in STEM research fields. Pursuing an academic career in the sciences requires persistence, perseverance, and courage. Despite awareness and implementation of measures to counteract them, women in STEM often face persistent challenges, including gender bias, stereotypes, unequal access to opportunities, and limited mentorship (Shen, 2013; Charlesworth and Banaji, 2019; Avolio et al., 2020; Jebsen et al., 2022; Freedman et al., 2023; Lathifa, 2023). Women juggle multiple roles in the workplace as researchers, teachers, and mentors, on top of administrative duties, and at home, women often shoulder much of the caregiving (Cech and Blair-Loy, 2019; Allen et al., 2023). The COVID-19 pandemic intensified these difficulties, adversely impacting work productivity, mental health, the pursuit of leadership positions, and an essential aspect for conducting outstanding research: achieving a healthy work-life balance (Gewin, 2020; Krukowski et al., 2021; National Academies of Sciences and Medicine, 2021; Lawson et al., 2023).

To highlight the remarkable contributions of female researchers in aquatic microbiology, we have curated a collection of articles in celebration of International Women's Day 2022. These articles encompass a wide range of research areas within aquatic microbiology, spanning diverse ecosystems, such as oceans and freshwater environments, and exploring microbiomes associated with rays and sharks. The studies delve into various aspects of aquatic microbial communities, including their response to climate change, the health of aquaculture animals, human health, and cellular enzymes.

Among the eight articles of this collection, Kim et al. investigated the effects of climate change on marine heterotrophic bacteria, which play an important role in ocean carbon cycling. The authors used mathematical modeling to demonstrate that bacterial biomass is sensitive to regional trends in temperature and organic carbon stocks. Their research focused on the surface and deep-water layers and provided a systematic global scale analysis.

A mini-review by Voskuhl and Rahlff summarized the current knowledge on natural and anthropogenic oil slicks as microbial habitats in the marine environment. Natural and anthropogenic surface slicks are widespread phenomena with ecosystem impacts. The slicks contribute to particle generation and carbon cycling, the accumulation of pollutants, and the dispersal of organisms. The authors emphasized the significance of studying microbial life within slicks, as they impact the composition, activity and interactions of microorganisms.

At a cellular level, Wang et al. explored the mechanisms of nitrate reductase (NR) in the toxic microalgae *Chattonella subsalsa* and found evidence for an ecological edge. Their research demonstrated the ability of *C. subsalsa* to differentially regulate NRs at multiple levels (transcriptional, translational, and post-translational levels) may allow this species to fine-tune nitrogen assimilation with photosynthetic activities in response to environmental cues. Their research suggests that the mechanism for this post-translational regulation was the reversible binding of 14-3-3 proteins, thus revealing a conserved mechanism between plants and algae that was previously unknown.

Two articles in this collection investigated the microbiomes of marine fishes (sharks and rays). Bregman et al. investigated two shark species in the Mediterranean Sea, the dusky shark (*Carcharhinus obscurus*) and the sandbar shark (*Carcharhinus plumbeus*). Their results revealed that not only do different organs contain specific microbiomes, but also these microbiomes are species-specific. The sampling, carried out over 3 years, also revealed differences in the abundance of a potential bacterial pathogen (*Streptococcus*) in the microbiomes across samples. Kerr et al. investigated the microbiomes on the epidermis of round rays (*Urobatis halleri*) and bat rays (*Myliobatis californica*) at two locations in Southern California. Their results show that the microbiome of the ray epidermis is species-specific, suggesting host selectivity. The bat rays (*M. californica*) produce high levels of protective dermal mucus and demonstrate high intra-species variability across their microbiomes. It is hypothesized that high mucus turnover may be a selective force driving their microbiome composition, much like occurs in the human gut. Furthermore, as the mucus supports high microbial growth rates, this may drive microbial competition and increase the antimicrobial properties of the microbes.

This collection of articles also covered freshwater environments. Ni et al. used the freshwater zebrafish (*Danio rerio*) as a model for investigating the potential of silver nanoparticles in aquaculture. They showed that low concentrations of silver nanoparticles promoted growth and caused no damage to the gills, intestines, or livers. In addition, silver nanoparticles upregulated the immune-regulated genes and modified the intestinal microflora. Some obligate and opportunistic pathogens significantly decreased in number in the intestinal microbiome, while beneficial bacteria capable of degrading pollutants and improving intestinal health significantly increased. These findings suggest that application of low concentrations of silver nanoparticles could positively impact the health of aquaculture animals. Ngo et al. studied another agricultural environment: paddy fields, essential ecosystems for humans, climate, and microbes. In this study, Ngo et al. isolated and identified cyanobacterial strains from paddy soil in Hanoi, Vietnam, and examined their cytotoxic activities. The authors emphasize the challenges associated with identifying cyanobacteria and stress the importance of using a polyphasic taxonomic approach for accurate identification. Interestingly, one particular *Scytonema* strain demonstrated potent cytotoxic activities against four human cell lines, suggesting that these organisms hold promise as potential sources of diverse and pharmacologically exciting compounds.

A mini-review by Bernasconi et al. focused on pit lakes, legacies of open-cut mining responsible for “ecosystem disservices”. The research on pit lakes has suffered from environmental stigma, a lack of interdisciplinary scientific collaboration, and unpublished data held by the mining industry. The authors provide insights into the microbial ecology of pit lakes, highlighting the importance of improving our understanding of microbial dynamics in these environments to ensure the safety of nearby communities.

In conclusion, the “*Women in aquatic microbiology: 2022*” highlights a large breadth of Research Topics led by women researchers around the globe, turning spotlight on their important contributions to the field.

## Author contributions

All authors listed have made a substantial, direct, and intellectual contribution to the work and approved it for publication.
